# Serum Cytokine Profile in IgA Nephropathy

**DOI:** 10.1016/j.xkme.2024.100940

**Published:** 2025-01-01

**Authors:** Friederike Selbach, Umberto Maggiore, Micaela Gentile, Kristin Meliambro, Kirk N. Campbell, Janusz Tucholski, Bruce A. Julian, William J. Placzek, Sara Alibrandi, Seunghee Kim-Schulze, Maria Lanau Martínez, M Loreto Fernandez-Lorente, Joaquin Manrique, Paolo Cravedi

**Affiliations:** 1 Department of Medicine, Translational Transplant Research Center (TTRC), Renal Division, Icahn School of Medicine at Mount Sinai, New York, NY; 2 UO Nefrologia, Dipartimento di Medicina e Chirurgia, Università di Parma, Parma, Italy; 3 Reliant Glycosciences, LLC, Birmingham, AL; 4 Human Immune Monitoring Center, Icahn School of Medicine at Mount Sinai, New York, NY; 5 Department of Nephrology, Hospital Universitario de Navarra, Pamplona, Spain

To the Editor:

Immunoglobulin A nephropathy (IgAN), the most common glomerulonephritis worldwide, leads to kidney failure in 20%-40% of cases over 20 years.^1^ Its pathogenesis involves a multi-step process starting with increased production of galactose-deficient IgA1 (Gd-IgA1), IgG formation against Gd-IgA1 (IgG anti-Gd-IgA1), and immune complex deposition that activates mesangial cells, causing glomerular injury and fibrosis.^2^

Because upper respiratory tract infections often trigger IgAN flares,^2^ we hypothesize that IgAN activity is associated with a specific proinflammatory cytokine fingerprint. To test this hypothesis, we used a large inflammation panel to decipher the cytokine profile of 53 IgAN patients and 19 controls. Detailed methods can be found in the Supplementary Material (Supplementary Methods).

Patients with IgAN and controls had similar ages (43.0 ± 16.0 years vs. 42.6 ± 16.7 years), with 54.7% versus 68.4% being male (Table 1). Most IgAN patients were White (45.3%), followed by Hispanic (18.9%), Asian (9.4%), and Black (5.7%). IgAN patients had moderate kidney function impairment with a median proteinuria of 1.8 (0.7-3.9) g/g urinary creatinine. Most patients (90.6%) were free of immunosuppression, and 60.8% were on renin-angiotensin-aldosterone system inhibitors.

**Table 1:** Population Characteristics

Characteristics	Controls (n = 19)	IgAN (n = 53)	*P*
Age, y	42.6 ± 16.7	43.0 ± 16.0	0.92
Male gender (%)	13 (68.4%)	29 (54.7%)	0.42
Ethnicity, n (%)^a^			
White	2 (25.0%)	24 (45.3%)	0.55
Black	1 (12.5%)	5 (9.4%)	
Hispanic	3 (37.5%)	10 (18.9%)	
Asian	2 (25.0%)	9 (17.0%)	
Other	0 (0.0%)	5 (9.4%)	
On immunosuppression, n (%)	N/A	5 (9.4%)	
On RAAS inhibitors, n (%)	N/A	31 (60.8%)	
Proteinuria, g/g urinary creatinine	N/A	1.8 (0.7-3.9)	
Serum creatinine, mg/dL	0.67 (0.60-0.87)^b^	1.26 (0.92-1.83)	0.002
eGFR, mL/min/1.73m^2^	115.8 ± 19.7^c^	67.9 ± 34.4	0.003
Serum albumin, g/dL	N/A	3.61 ± 0.74	
Hematuria, RBC/uL	N/A	30 (20-100)	
MEST-C score, value	N/A	1.73 ± 1.58^d^	
M in MEST-C score		0.36 ± 0.49	
M0, n (%)		14 (63.6%)	
M1, n (%)		8 (36.4%)	
E in MEST-C score		0.55 ± 0.51	
E0, n (%)		10 (45.5%)	
E1, n (%)		12 (54.5%)	
S in MEST-C score		0.14 ± 0.35	
S0, n (%)		19 (86.4%)	
S1, n (%)		3 (13.6 %)	
T in MEST-C score		0.36 ± 0.58	
T0, n (%)		15 (68.2%)	
T1, n (%)		6 (27.3%)	
T2, n (%)		1 (4.5%)	
C in MEST-C score		0.32 ± 0.57	
C0, n (%)		16 (72.7%)	
C1, n (%)		5 (22.7%)	
C2, n (%)		1 (4.5 %)	

*Note:* Mann-Whitney test for continuous variables (reported as mean ± standard deviation, or median [interquartile range]), Fisher exact test for categorical variable.

Abbreviations: IgAN, Immunoglobulin A nephropathy.

a Data available for 8 controls.

b Data available for 7 controls.

c Data available for 6 controls.

d Data from 22 IgAN patients.

The serum levels of total IgA (5282 [IQR: 7,062-3,016] μg/mL vs 2,709 [3,826-1,618] μg/mL; *P* < 0.001), Gd-IgA1 (10 [14.6-6.2] μg/mL vs 6.2 [7.9-4.1] μg/mL; *P* = 0.01), and IgG anti-Gd-IgA1 autoantibodies (0.59 [0.81-0.48] vs 0.51 [0.6-0.4] μg/mL; *P* = 0.01) were significantly higher in patients with IgAN than controls (Fig. S1). IgA and Gd-IgA1 were significantly correlated (rho = 0.364; *P* = 0.01) and both correlated positively with serum creatinine (rho = 0.405; *P* = 0.005 and rho = 0.428; *P* = 0.003, respectively) and negatively with estimated glomerular filtration rate (eGFR) (rho = −0.339; *P* = 0.02 and rho = −0.356; *P* = 0.01, respectively), whereas IgG anti-Gd-IgA1 showed no correlation.

Overall, cytokine levels were higher in IgAN patients than controls, and 16 of them were significantly different between the groups (Tables S1-S3). To determine the smallest subset of cytokines that could jointly discriminate between IgAN and controls, we used the adaptive LASSO approach for logistic regression, which identified the cytokines CX3CL1, IL-12β, and IL-6 (Fig. S2).

Pairs of these cytokines already separated IgAN patients from controls well (Fig. 1A), and separation between the 2 groups improved further in 3-dimensional scatter plots based on CX3CL1, IL-12β, and IL-6 combined (Fig. 1B). The combined use of CX3CL1, IL-12β, and IL-6 increased the area under the receiver operating characteristic curve up to 0.95 when compared with considering each cytokine in isolation (Fig. 1C). Model sensitivity was 96.2% and specificity 73.7%. The model could correctly classify 90.3% of the individuals as IgAN patients versus controls. Cross-validated mean area under the receiver operating characteristic curve of the logistic model based on CX3CL1, IL-12β, and IL-6 (see multiple logistic regression coefficients in Table S4) decreased only slightly from 0.95 to 0.912 (bootstrap bias corrected 95% CI: 0.808-0.975).

**Figure 1:**
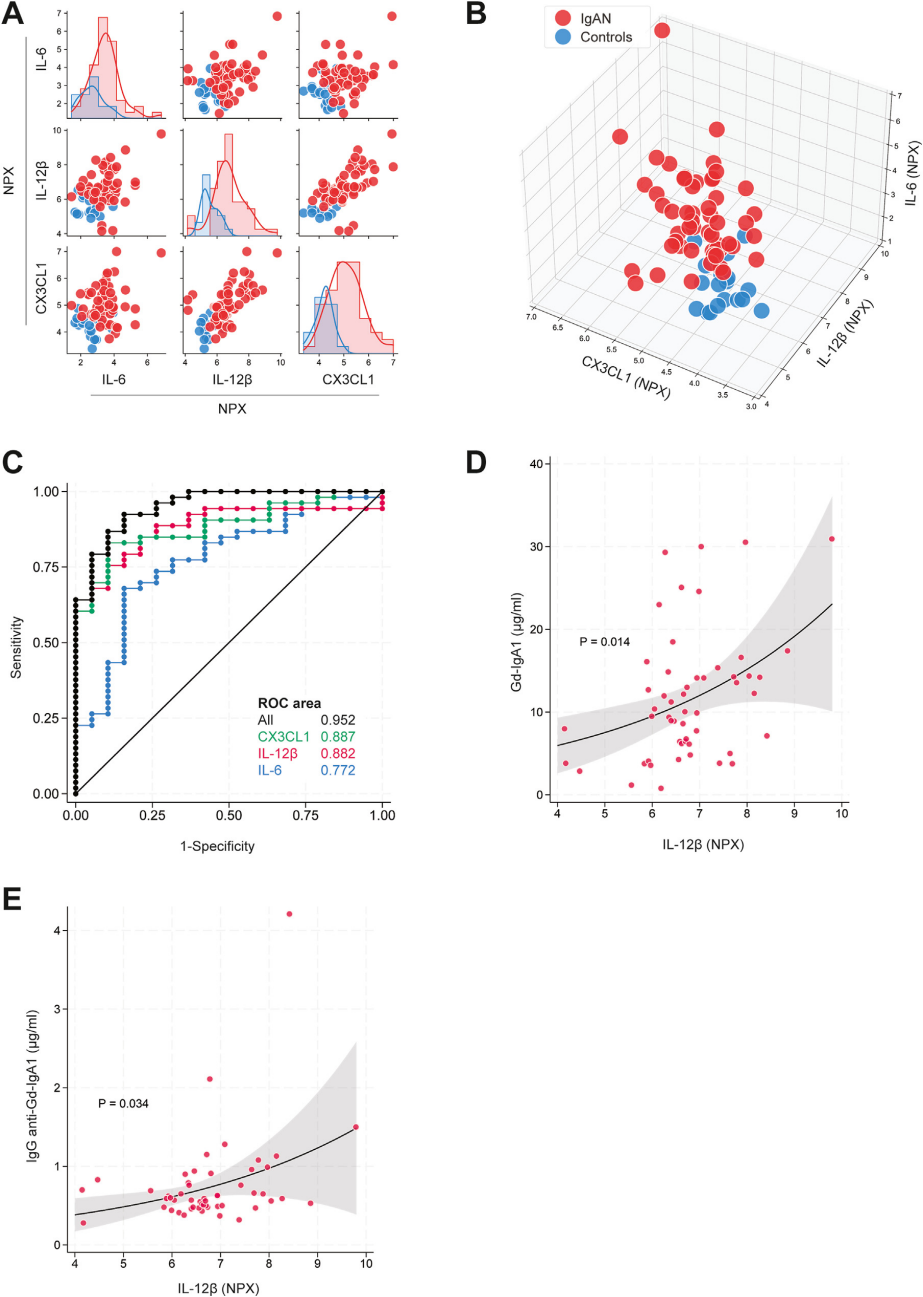
CX3CL1, IL-12β, and IL-6 differentiate patients with IgAN from controls and correlate with antibodies. (A) Matrix of pairwise scatter plots, and overlaying histograms with kernel density line of IgAN patients (red) and controls (blue) of the selected covariates CX3CL1, IL-12β, and IL-6 that adaptive LASSO selected for best prediction of IgAN or control membership. The scatter plots off the diagonal, and the histogram/kernel density line along the diagonal assist to visually identify which bivariate associations among the 3 cytokines separate the 2 patient groups the most. (B) Three-dimensional scatter plots of the selected cytokines. (C) The ROC curves from logistic regression model of CX3CL1, IL-12β, and IL-6 in isolation and combined; (D-E) Gamma multiple regression model indicates significant association for IL-12β with (D) Gd-IgA1 (*P* = 0.014) and (E) IgG anti-Gd-IgA1 (*P* = 0.034). The black line represents fitted values of (D) Gd-IgA1 and (E) IgG anti-Gd-IgA1 as a function of IL-12β from gamma multiple regression models that also included CX3CL1 and IL-6 among the independent covariates. The scatterplot of actual data points (red circles) is superimposed. Shaded areas represent 95 % CIs. NPX, normalized protein expression; ROC, receiver operating characteristic.

The CX3CL1 was significantly associated with proteinuria (rho = 0.408; *P* = 0.003), low eGFR (rho = −0.647; *P* < 0.001), and low serum albumin levels (rho = −0.275; *P* = 0.05) (Fig. S3). The IL-12β was associated with low eGFR (rho = −0.576; *P* < 0.001, Fig. S3), and IL-6 was associated with low serum albumin (rho = −0.394; *P* = 0.004, Fig. S3). None of the 3 cytokines showed a statistically significant correlation with hematuria.

The multivariable regression model that jointly included CX3CL1, IL-12β, and IL-6, demonstrated that IL-12β was associated with increased levels of circulating Gd-IgA1 (*P* = 0.01, Fig. 1D) and with increased levels of IgG anti-Gd-IgA1 autoantibodies (*P* = 0.03, Fig. 1E), whereas there was no significant association between CX3CL1 or IL-6 and serum IgA, Gd-IgA1, or IgG anti-Gd-IgA1.

Our comprehensive analysis showed that IgAN patients exhibit a significant increase of numerous serum cytokines, including those involved in B cell signaling, IgA production, immune regulation, macrophage activation, and tissue remodeling. Our analyses unveiled that the combination of CX3CL1, IL-12β, and IL-6 is particularly effective in differentiating IgAN patients from controls. Previous studies linked CX3CL1-CX3CR1 signaling to acute and chronic kidney disease and reported elevated levels of IL-6 and IL-12 in chronic kidney disease. This study newly showed that levels of CX3CL1, IL-12β, and IL-6 are associated with signs of disease severity in IgAN patients. Of importance, IL-12β was significantly associated with increased levels of circulating Gd-IgA1 and IgG anti-Gd-IgA1 autoantibodies, suggesting a mechanistic link.

CX3CL1 (or fractalkine) is a chemokine and adhesion molecule,^3^ playing a key pathogenic role in inflammatory glomerular diseases. Our data confirm and expand previous evidence from a Chinese cohort of IgAN patients indicating that circulating CX3CL1 levels correlate with disease severity and associate with kidney B cell and macrophage infiltration and mesangial hypercellularity.^4^

IL-12 consists of 2 subunits, α and β, and is primarily produced by monocytes and macrophages.^5^ Of note, IL-12β can also partner with subunit α p19 and form the cytokine IL-23, which is strongly implicated in autoimmunity.^6^ In vitro, IL-12β induces gene expression of APRIL (A Proliferation-Inducing Ligand), a B cell-activating factor that promotes Gd-IgA1 production.^7^ Remarkably, our data unraveled an association between IL-12β and high Gd-IgA1 and IgG anti-Gd-IgA1 levels in IgAN patients. As these antibodies play a critical pathogenic role, our data support the testable hypothesis that targeting IL-12β improves disease outcomes.

IL-6 has been previously reported to be associated with the risk of IgAN development^8^ and progression.^9^ This cytokine counteracts IgA galactosylation by B cells, leading to increased Gd-IgA1 production and is secreted by mesangial cells on IgA1 deposition.^8^ Together with CX3CL1 and IL-12β, IL-6 may represent a key inflammatory cytokine mediating the association between airway infections and IgAN relapses.

A limitation of previous studies was the narrow number of cytokines tested. Despite the relatively small sample size, our large cytokine panel allowed us to unravel that combination of CX3CL1, IL-12β, and IL-6 identified IgAN patients better than each of these cytokines taken singularly. If validated, this information may lead to biomarker discovery and to the identification of molecular pathways implicated in IgAN pathophysiology.

## Supplementary Materials

Supplementary File (PDF) Figs S1-S3; Item S1; Table S1-S4.
