# Supplementary material for: Serum Cytokine Profile in IgA Nephropathy
Source: Kidney Med. 2025 Jan 1;7(2):100940. doi: 10.1016/j.xkme.2024.100940 (PMC11751519; doi:10.1016/j.xkme.2024.100940)
Supplement: Supplementary File (PDF) — Figs S1-S3; Item S1; Table S1-S4. [file mmc1.pdf]

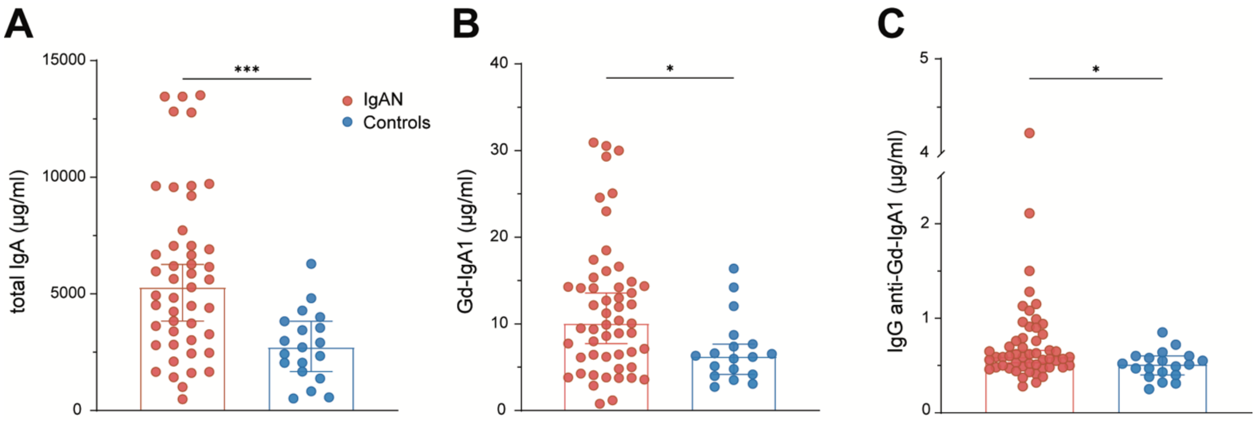

Figure S1. Levels of IgA, Gd-IgA1, and IgG anti-Gd-IgA1 in patients with IgAN and controls. Serum levels of (**A**) total IgA, (**B**) Gd-IgA1, and (**C**) IgG anti-Gd-IgA1 in IgAN patients (red) and controls (blue); *P<0.05; ***P<0.001, Mann-Whitney test. IgA, immunoglobulin A; IgAN, IgA nephropathy; Gd-IgA1, galactose-deficient immunoglobulin A1; IgG anti-Gd-IgA1, Immunoglobulin G anti-GdIgA1. Data are expressed as mean ± standard deviation. Total IgA and Gd-IgA1 data were missing for 6 and 1 study participants, respectively.

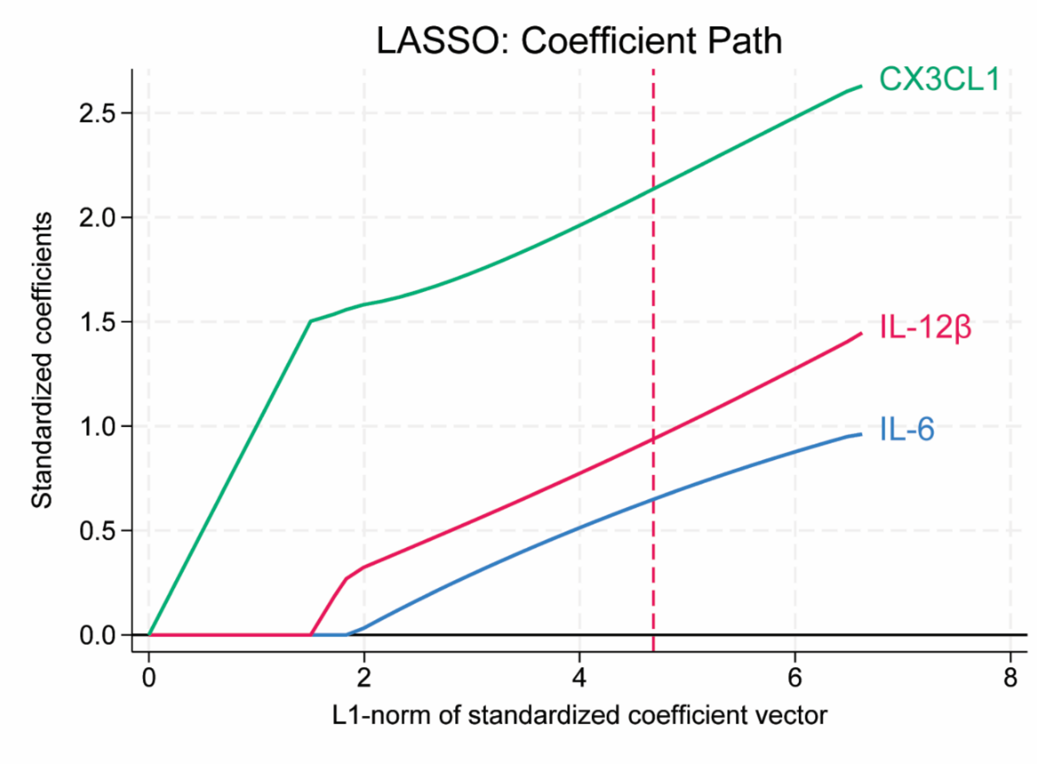

Figure S2. Variables characterizing IgAN patients according to adaptive LASSO for analyte selection via logistic regression for discriminating between IgAN patients and controls. The plot shows standardized coefficient estimates (y-axis) as a function of the 'L1-norm' of the standardized analyte coefficients (i.e. the maximum allowed sum of the absolute values of the coefficients) (x-axis). The tuning parameter (not shown in the plot) 'shrinks' the coefficient toward zero as its value increases. By setting some coefficients to zero, the tuning parameter determines which variables (analytes) the LASSO will eventually exclude. The final value of the L1-norm (vertical dash red line) resulted from the selection among the potential candidates of the tuning parameters that were estimated by cross-validation. The final value of the selected analyte coefficients resulted from the projection on the y-axis of the intersection between the vertical dash red line (i.e., the final value of the L1-norm) and the solid color lines.

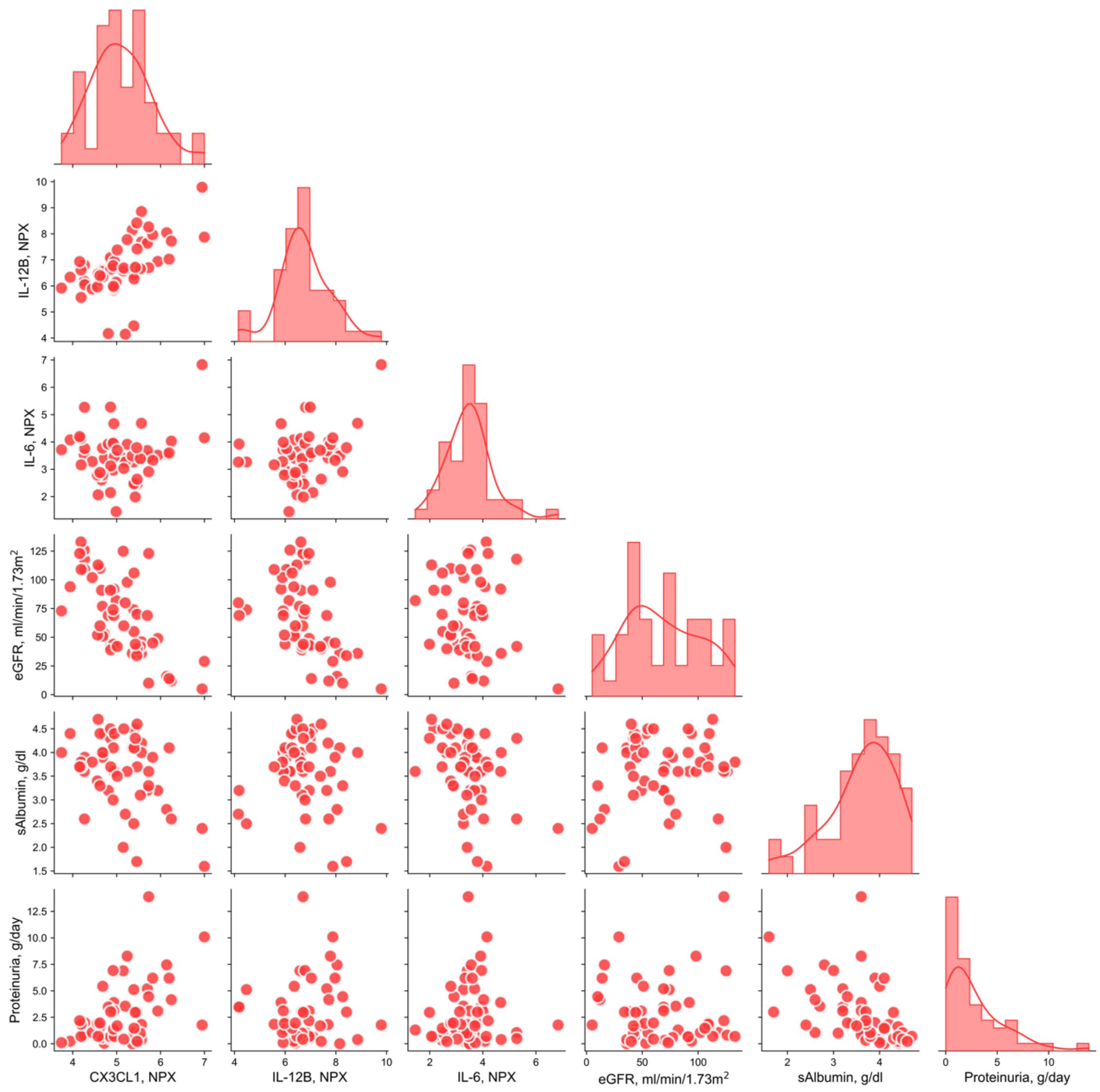

Figure S3 Grid of paired scatter plots between the selected cytokines (CX3CL1, IL12B, and IL-6) and eGFR, serum albumin (sAlbumin), and proteinuria. Off-diagonal plots are the paired scatterplots; diagonal plots represent variable distribution. CX3CL1 was significantly associated with proteinuria (rho=0.408; P=0.003), low eGFR (rho= -0.647; P<0.001), and low serum albumin levels (rho= -0.275; P=0.05); IL-12β was associated with low eGFR (rho= -0.576; P<0.001); IL-6 was associated with low serum albumin (rho= -0.394 P=0.004)

Item S1: SUPPLEMENTARY METHODS

Patients

A total of 53 IgAN patients and 19 healthy controls were enrolled at the Hospital Universitario de Navarra, Pamplona, Spain, (10 healthy controls, 19 IgAN patients), and the Mount Sinai Hospital, New York, NY, USA, (9 healthy controls, 34 IgAN patients) under institutional review board's approvals (Approval Pyto201793 and IRB STUDY 14-00700, respectively). Serum samples from patients with IgAN were collected on the day of the kidney biopsy, before the procedure as part of the institutional biobanks. IgAN patients were diagnosed via histopathology of kidney biopsy specimen. Controls were identified as healthy blood donors at both institutions. Written informed consent was obtained from all individuals prior to participation. The study adhered to the Declaration of Helsinki Principles.

Serum isolation and analysis

The protocol for blood collection, serum isolation and storage were similar between the two institutions. Serum was processed within 2 hours from collection to avoid cytokine degradation and stored at -20C. Serum analysis was performed for all samples collectively at Mt. Sinai Hospital and Reliant Glycosciences, LLC, Birmingham, Alabama. The investigators performing serum analyses were blinded to the clinical information. More details about the performance of the Olink assay are provided here (S1).

Measurement of serum levels of IgA, Gd-IgA1, and IgG anti-Gd-IgA1 autoantibodies

For the quantification of total serum IgA and serum Gd-IgA1 we employed a commercially available enzyme-linked immunosorbent assay (ELISA) test kit (ThermoFisher Scientific and IBL America, respectively) and followed the manufacturer's protocol. Measurement of serum IgG anti-Gd-IgA1 autoantibody levels was performed by RELIANT GLYCOSCIENCES, LLC, using an ELISA based approach with a Gd-IgA1 capture reagent as the antigen.

Serum analyte quantification

Serum inflammatory markers were assessed using the Olink® Target 96 Inflammation panel (**Supplementary Table S2,** 92 analytes), a commercially available product from Olink Proteomics in Uppsala, Sweden. This panel employs Proximity Extension Assay (PEA) technology, a multiplex immunoassay that utilizes target-specific antibodies and oligonucleotide binding, followed by quantitative real-time polymerase chain reaction (qPCR) for the measurement of selected analytes (S2). PEA allows for robust analysis on very small sample volumes while maintaining a high degree of specificity and sensitivity (S3). This approach appears to mitigate technical challenges such as antibody cross-reactivity and inter-assay variability commonly observed in multiplex ELISA (S4).

The resulting threshold cycle (Ct) data underwent quality control and normalization procedures utilizing internal and external controls. The final assay read-out is presented as Normalized Protein Expression (NPX) values, obtained after normalization between runs by subtracting the corresponding dCq-value for the Interplate Control (IPC) from

the generated dCq-values. NPX values are then adjusted relative to a correction factor determined by Olink and are represented on a log2 scale, where higher numbers indicate higher protein levels in the sample.

14 analytes did not pass quality control and were excluded from analysis (**Supplementary Table S3**). Three samples (two IgAN patients and one healthy control) did not meet quality control criteria and were also excluded from analysis.

Statistical Analysis

All analyses were performed using Stata 18 (2023, StataCorp, College Station TX, USA) and Python 3.10 (URL https://www.python.org/). A two-sided P value of less than 0.05 was regarded as statistically significant. The differences between groups were tested in continuous variables using the Mann-Whitney test, and in categorical variables using Fisher’s exact test, and the relationship between continuous variables with Spearman's rank correlation coefficient. With regard to the univariate differences between groups in the 78 analytes, we used the Holm multiple-testing procedure to control the type I error (S5). Because several analytes conveyed redundant information, we aimed at selecting, among the several statistically significant ones, the small subset of analytes that jointly discriminated between IgAN patients and controls, by fitting a logistic model. For this purpose, we used Adaptive LASSO (least absolute shrinkage and selection operator) for logistic regression (S6). We estimated AUCROC curve associated with the selected logistic model after 10-fold cross-validation, and calculated Bootstrap Corrected 95 percent confidence intervals. Finally, we examined the linear relation between the jointly selected analytes and serum levels of IgA, Gd-IgA1, and IgG anti-Gd-IgA1 autoantibodies in patients with IgAN. To this purpose, we used gamma

regression via generalized multiple regression models with robust standard errors with log link due to the non-normal distribution with long tight tails of serum Ig levels. For each model, the dependent variable was the serum levels of IgA, Gd-IgA1, and IgG anti-Gd-IgA1 autoantibodies, and the independent variables were the jointly selected analytes.

Additional information: Adaptive LASSO

We used LASSO for logistic regression to make the selection among the statistically significant analytes of those that helped in discriminating between IgAN and controls. LASSO is meant for high-dimensional models having too many potential covariates possibly highly correlated for the sample size at hand. It is meant to avoid overfitting the dataset by preventing the inclusion of spurious predictors that would likely not be confirmed upon validation in an external dataset. LASSO is a procedure for selecting covariates: it does not provide P-values. LASSO estimates coefficients of the regression model as a function of a tuning parameter that ‘shrinks’ the coefficient toward zero as its value gets larger. In other words, the value of the tuning parameter acts as a volume knob: large values of the parameter penalize model complexity (i.e., many analytes predicting IgAN membership), whereas smaller values weakly penalize model complexity. By setting a coefficient to zero, the tuning parameter determines which analytes the LASSO will eventually exclude. The potential values of the tuning parameter cannot be estimated by the data; therefore, their candidate values need to be calculated by cross-validation. Among the possible LASSO algorithms, we chose the adaptive LASSO. Adaptive LASSO consists of multiple LASSO steps. Each LASSO step is based on cross-validation; variables with a zero coefficient are discarded after

each successive LASSO. The algorithm is based on weights that are designed to drive small coefficient estimates to zero in the next step. We chose adaptive LASSO since it provides better selection and bias reduction compared to standard LASSO (S7, S8). The plot reporting the instance when LASSO selected the variable in this model is reported below (**Figure S1**). The Stata and Python code for all analyses is freely available at https://github.com/UMaggiore/Cytokines-and-IgAN; the dataset is available upon request.

Table S1. Difference in analytes between groups.

Analyte	Control	IgAN	P value	Adj. P value
**CX3CL1**	**4.2 ± 0.4**	**5.1 ± 0.7**	**<0.001**	**0.000051**
**IL-12β**	**5.6 ± 0.5**	**6.8 ± 1.0**	**<0.001**	**0.000069**
**CD5**	**4.9 ± 0.3**	**5.6 ± 0.5**	**<0.001**	**0.000073**
**TNFRSF9**	**5.6 ± 0.5**	**6.7 ± 0.9**	**<0.001**	**0.000093**
**CSF-1**	**9.0 ± 0.2**	**9.4 ± 0.2**	**<0.001**	**0.000144**
**PDL-1**	**5.4 ± 0.5**	**6.2 ± 0.6**	**<0.001**	**0.000319**
**IL-15RA**	**0.7 ± 0.2**	**1.4 ± 0.6**	**<0.001**	**0.000377**
**IL-10RB**	**5.6 ± 0.3**	**6.1 ± 0.3**	**<0.001**	**0.000420**
**FGF-23**	**0.3 ± 0.5**	**1.2 ± 1.1**	**<0.001**	**0.000951**
**CCL3**	**6.1 ± 0.7**	**7.1 ± 1.4**	**<0.001**	**0.004271**
**CCL4**	**6.3 ± 0.4**	**6.9 ± 0.6**	**<0.001**	**0.006803**
**MMP-10**	**8.2 ± 0.7**	**8.9 ± 0.5**	**<0.001**	**0.007445**
**TNF**	**3.4 ± 0.5**	**4.0 ± 0.6**	**<0.001**	**0.007728**
**CD40**	**10.5 ± 0.5**	**11.1 ± 0.7**	**<0.001**	**0.008446**
**IL-6**	**2.7 ± 0.7**	**3.5 ± 0.9**	**<0.001**	**0.030409**
**CST5**	**7.0 ± 0.7**	**7.5 ± 0.6**	**<0.001**	**0.036206**
IL-18	8.5 ± 0.8	9.1 ± 0.6	0.001	0.074405
CCL23	10.8 ± 0.5	11.3 ± 0.5	0.001	0.076553
TWEAK	9.4 ± 0.5	9.1 ± 0.3	0.001	0.078729
SLAMF1	2.1 ± 0.5	2.6 ± 0.6	0.001	0.088410
FGF-21	3.9 ± 1.5	5.4 ± 1.5	0.002	0.099117
IL-17C	2.2 ± 1.1	3.1 ± 1.0	0.002	0.106245
CCL19	9.5 ± 0.6	10.2 ± 1.0	0.002	0.108988
IL-10	2.8 ± 0.7	3.4 ± 0.8	0.002	0.116647
4E-BP1	5.6 ± 0.8	6.5 ± 1.1	0.003	0.135753
IFNγ	6.5 ± 0.6	7.1 ± 1.0	0.004	0.185846
VEGFA	11.8 ± 0.5	12.2 ± 0.4	0.007	0.372141
SCF	8.5 ± 0.3	8.7 ± 0.4	0.01	0.531218
CD8A	9.4 ± 0.6	9.8 ± 0.8	0.01	0.581173
FGF-5	1.2 ± 0.3	1.4 ± 0.4	0.01	0.657870
STAMBP	3.6 ± 0.4	4.0 ± 0.7	0.02	0.883656
IL-20RA	0.0 ± 0.2	0.3 ± 0.5	0.02	0.991752
CXCL9	5.8 ± 0.7	6.5 ± 1.3	0.04	> 0.99
IL-18R1	6.4 ± 0.4	6.7 ± 0.5	0.04	> 0.99
CCL25	5.8 ± 0.5	6.1 ± 0.6	0.04	> 0.99
GDNF	1.3 ± 0.4	1.6 ± 0.5	0.05	> 0.99
MCP-4	14.9 ± 0.6	14.5 ± 1.0	0.05	> 0.99
MMP-1	14.1 ± 0.6	14.3 ± 0.7	0.05	> 0.99
SIRT2	2.0 ± 0.8	2.5 ± 1.1	0.05	> 0.99
OSM	6.1 ± 0.8	5.5 ± 1.2	0.05	> 0.99
UPA	9.5 ± 0.3	9.3 ± 0.4	0.07	> 0.99
EN-RAGE	5.7 ± 0.7	5.2 ± 1.2	0.07	> 0.99
DNER	8.4 ± 0.3	8.3 ± 0.3	0.08	> 0.99
CASP-8	2.8 ± 0.6	3.1 ± 0.8	0.09	> 0.99
LAP_TGFβ1	7.8 ± 0.4	7.5 ± 0.6	0.10	> 0.99
CCL28	1.9 ± 0.5	1.7 ± 0.7	0.10	> 0.99
NT3	2.2 ± 0.4	2.1 ± 0.6	0.10	> 0.99
CXCL1	8.5 ± 0.5	8.8 ± 0.7	0.15	> 0.99
CXCL11	7.1 ± 0.8	7.5 ± 1.2	0.15	> 0.99

Analyte	Control	IgAN	P value	Adj. P value
CXCL6	8.1 ± 0.6	8.4 ± 0.7	0.16	> 0.99
CDCP1	3.1 ± 0.9	3.2 ± 0.8	0.17	> 0.99
MCP-3	1.9 ± 0.9	2.0 ± 0.7	0.19	> 0.99
TNFSF14	6.1 ± 0.7	5.7 ± 1.0	0.21	> 0.99
LIF-R	2.9 ± 0.2	3.1 ± 0.4	0.23	> 0.99
AXIN1	2.5 ± 0.7	2.7 ± 0.9	0.23	> 0.99
IL-8	6.3 ± 0.5	6.6 ± 0.9	0.25	> 0.99
CCL20	6.9 ± 1.7	7.3 ± 1.0	0.28	> 0.99
TGF-α	4.4 ± 0.7	4.2 ± 0.9	0.29	> 0.99
IL-10RA	0.7 ± 0.6	0.7 ± 0.8	0.34	> 0.99
CXCL10	7.3 ± 0.7	7.5 ± 1.1	0.44	> 0.99
MCP-1	11.5 ± 0.4	11.5 ± 0.5	0.46	> 0.99
CXCL5	11.9 ± 0.5	11.8 ± 1.0	0.46	> 0.99
ADA	5.2 ± 0.4	5.1 ± 0.4	0.46	> 0.99
CCL11	7.4 ± 0.6	7.5 ± 0.6	0.54	> 0.99
IL-2Rβ	1.0 ± 0.4	1.1 ± 0.7	0.56	> 0.99
TNFβ	4.0 ± 0.4	4.0 ± 0.6	0.58	> 0.99
FGF-19	8.5 ± 1.1	8.6 ± 0.9	0.68	> 0.99
FIT3L	8.8 ± 0.4	8.9 ± 0.6	0.72	> 0.99
HGF	8.5 ± 0.5	8.4 ± 0.6	0.72	> 0.99
TRAIL	8.2 ± 0.3	8.3 ± 0.3	0.73	> 0.99
MCP-2	8.9 ± 0.9	8.9 ± 0.8	0.74	> 0.99
CD6	5.3 ± 0.4	5.3 ± 0.5	0.75	> 0.99
TRANCE	5.1 ± 0.8	5.2 ± 0.7	0.78	> 0.99
ST1A1	3.0 ± 1.1	2.9 ± 1.4	0.82	> 0.99
IL-17A	0.5 ± 0.9	0.5 ± 0.9	0.86	> 0.99
CD244	5.2 ± 0.4	5.2 ± 0.4	0.91	> 0.99
IL-7	2.1 ± 0.5	2.1 ± 0.6	0.95	> 0.99
OPG	9.1 ± 0.4	9.1 ± 0.5	0.98	> 0.99

Data are reported as mean ± standard deviation. Analytes are ranked by statistical significance and expressed as Normalized Protein eXpression (NPX). P value, nominal P values by Mann-Whitney test. Adj. P val., Adjusted P values after Holm correction for multiple tests. An adjusted P value of less than 0.05 was regarded as statistically significant.

Table S2. Analyte essay.

Adenosine Deaminase (ADA)	Hepatocyte growth factor (HGF)	Monocyte chemotactic protein 1 (MCP-1)
Artemin (ARTN)	Interferon gamma (IFN-gamma)	Monocyte chemotactic protein 2 (MCP-2)
Axin-1 (AXIN1)	Interleukin-1 alpha (IL-1 alpha)	Monocyte chemotactic protein 3 (MCP-3)
Beta-nerve growth factor (Beta-NGF)	Interleukin-2 (IL-2)	Monocyte chemotactic protein 4 (MCP-4)
Caspase-8 (CASP-8)	Interleukin-2 receptor subunit beta (IL-2RB)	Natural killer cell receptor 2B4 (CD244)
C-C motif chemokine 3 (CCL3)	Interleukin-4 (IL-4)	Neurotrophin-3 (NT-3)
C-C motif chemokine 4 (CCL4)	Interleukin-5 (IL5)	Neurturin (NRTN)
C-C motif chemokine 19 (CCL19)	Interleukin-6 (IL6)	Oncostatin-M (OSM)
C-C motif chemokine 20 (CCL20)	Interleukin-7 (IL-7)	Osteoprotegerin (OPG)
C-C motif chemokine 23 (CCL23)	Interleukin-8 (IL-8)	Programmed cell death 1 ligand 1 (PD-L1)
C-C motif chemokine 25 (CCL25)	Interleukin-10 (IL10)	Protein S100-A12 (EN-RAGE)
C-C motif chemokine 28 (CCL28)	Interleukin-10 receptor subunit alpha (IL-10RA)	Signaling lymphocytic activation molecule (SLAMF1)
CD40L receptor (CD40)	Interleukin-10 receptor subunit beta (IL-10RB)	SIR2-like protein 2 (SIRT2)
CUB domain-containing protein 1 (CDCP1)	Interleukin-12 subunit beta (IL-12B)	STAM-binding protein (STAMBP)
C-X-C motif chemokine 1 (CXCL1)	Interleukin-13 (IL-13)	Stem cell factor (SCF)
C-X-C motif chemokine 5 (CXCL5)	Interleukin-15 receptor subunit alpha (IL-15RA)	Sulfotransferase 1A1 (ST1A1)
C-X-C motif chemokine 6 (CXCL6)	Interleukin-17A (IL-17A)	T cell surface glycoprotein CD6 isoform (CD6)
C-X-C motif chemokine 9 (CXCL9)	Interleukin-17C (IL-17C)	T-cell surface glycoprotein CD5 (CD5)
C-X-C motif chemokine 10 (CXCL10)	Interleukin-18 (IL-18)	T-cell surface glycoprotein CD8 alpha chain (CD8A)
C-X-C motif chemokine 11 (CXCL11)	Interleukin-18 receptor 1 (IL-18R1)	Thymic stromal lymphopoietin (TSLP)
Cystatin D (CST5)	Interleukin-20 (IL-20)	TNF-beta (TNFB)
Delta and Notch-like epidermal growth factor-related receptor (DNER)	Interleukin-20 receptor subunit alpha (IL-20RA)	TNF-related activation-induced cytokine (TRANCE)
Eotaxin (CCL11)	Interleukin-22 receptor subunit alpha-1 (IL-22 RA1)	TNF-related apoptosis-inducing ligand (TRAIL)
Eukaryotic translation initiation factor 4E-binding protein 1 (4E-BP1)	Interleukin-24 (IL-24)	Transforming growth factor alpha (TGF-alpha)
Fibroblast growth factor 21 (FGF-21)	Interleukin-33 (IL-33)	Tumor necrosis factor (Ligand) superfamily, member 12 (TWEAK)
Fibroblast growth factor 23 (FGF-23)	Latency-associated peptide transforming growth factor beta-1 (LAP TGF-beta-1)	Tumor necrosis factor (TNF)
Fibroblast growth factor 5 (FGF-5)	Leukemia inhibitory factor (LIF)	Tumor necrosis factor ligand superfamily member 14 (TNFSF14)
Fibroblast growth factor 19 (FGF-19)	Leukemia inhibitory factor receptor (LIF-R)	Tumor necrosis factor receptor superfamily member 9 (TNFRSF9)
Fms-related tyrosine kinase 3 ligand (Flt3L)	Macrophage colony-stimulating factor 1 (CSF-1)	Urokinase-type plasminogen activator (uPA)
Fractalkine (CX3CL1)	Matrix metalloproteinase-1 (MMP-1)	Vascular endothelial growth factor A (VEGF-A)

Glial cell line-derived neurotrophic factor (GDNF)	Matrix metalloproteinase-10 (MMP-10)	

Table S3. Analytes below quality control.

ARTN	IL-20	IL-5
Beta-NGF	IL-22 RA1	LIF
IL-1 alpha	IL-24	NRTN
IL-13	IL-33	TSLP
IL-2	IL-4	

Table S4. Final 3-parameter logistic regression model (fitted without LASSO penalization).

Parameter	Coefficient [95 percent confidence interval]
**IL-6**	1.70** [0.47, 2.93]
**IL-12β**	1.61* [0.11, 3.11]
**CX3CL1**	3.15** [1.13, 5.16]
**Intercept**	3.57*** [1.68, 5.47]
**AIC**	41.58
**BIC**	50.69
**Pseudo R2**	0.60

*** p<.001, ** p<.01, * p<.05.
The final 3-parameter model was locked as
XB= 3.57 + (1.70*IL-6) + (1.61*IL-12β) + (3.15*CX3CL1).
Where the X variables are transformed as follows:
IL-6 = (IL-6 – 3.25) / 0.91;
IL-12β = (IL-12β – 6.44) / 1.07;
CX3CL1 = (CX3CL1 – 4.87) / 0.74.
The probability of IgAN is derived as the P= $1/(1+e^{-XB})$.
LASSO penalized coefficients are not reported as they only serve for variable selection purposes
AIC, Akaike information criterion; BIC, Bayesian information criterion.

SUPPLEMENTARY REFERENCES

S1. Carlyle, B.C., Kitchen R.R., Celia A.M., et al. Technical Performance Evaluation of Olink Proximity Extension Assay for Blood-Based Biomarker Discovery in Longitudinal Studies of Alzheimer's Disease. *Front Neurol*, 13:889647 (2022).

S2. Lundberg, M., Eriksson, A., Tran, B., Assarsson, E. & Fredriksson, S. Homogeneous antibody-based proximity extension assays provide sensitive and specific detection of low-abundant proteins in human blood. (2011) doi:10.1093/nar/gkr424.

S3. Assarsson, E. *et al.* Homogenous 96-plex PEA immunoassay exhibiting high sensitivity, specificity, and excellent scalability. *PLoS One* **9**, (2014).

S4. Tighe, P. J., Ryder, R. R., Todd, I. & Fairclough, L. C. ELISA in the multiplex era: potentials and pitfalls. *Proteomics Clin Appl* **9**, 406–422 (2015).

S5. Wasserman, L. & Roeder, K. High-dimensional variable selection. *https://doi.org/10.1214/08-AOS646* **37**, 2178–2201 (2009).

S6. Zou, H. The Adaptive Lasso and Its Oracle Properties. (2006) doi:10.1198/016214506000000735.

S7. Sture Holm. A Simple Sequentially Rejective Multiple Test Procedure. *Scand J Statist* **6**, 65–70 (1979).

S8. Freijeiro-González, L., Febrero-Bande, M. & González-Manteiga, W. A Critical Review of LASSO and Its Derivatives for Variable Selection Under Dependence Among Covariates. *International Statistical Review* **90**, 118–145 (2022).
